# Design approach of perforated labyrinth-based acoustic metasurface for selective acoustic levitation manipulation

**DOI:** 10.1038/s41598-021-87179-x

**Published:** 2021-04-07

**Authors:** Zhike Xu, Ling Qin, Wei Xu, Shuhua Fang, Jiyao Wang

**Affiliations:** 1 School of Electrical Engineering, Southeast University, Nanjing, Jiangsu China; 2 Ford Motor Company, Dearborn, MI USA

**Keywords:** Mechanical engineering, Acoustics

## Abstract

This paper proposes a metasurface design approach with perforated labyrinthine path coil structure to manipulate the acoustic transmission with inexpensive materials. The medium in the labyrinthine path coils in this design is air, but not limited to air. A systematic approach has been proposed for the unit cell design of acoustic metamaterials with adjustable resonance peak frequencies and bandgap width. The theory demonstrates that the length of pipe segments determines resonance peak frequencies and the cross-sectional area ratio adjusts the bandgap width. The proposed design approach uses an equivalent pipe circuit based analytical model to design the high transmission (high pass) and high reflection (low pass) unit cell. The simulation and experiment has been performed to evaluate the validity of the theory. Although there exists some assumptions in the theory, the theory still has enough accuracy to guide the metasurface design illustrated by the simulation and experiment results.

## Introduction

Over the last three decades, there has been a growing interest in acoustic metamaterials. The active investigation of the photonic crystals gradually leads to relative mature theoretical prediction and subsequent experimental validation of photonic bandgap structures^1–3^. Similar studies on acoustic phononic crystals and their bandgap structures have been conducted both theoretically and experimentally^4–7^. Similar as the optical counterpart, these acoustic metamaterials give rise to spatial compression of wave energy, wave signal buffering, invisible acoustic cloaking, noise mitigation and nonlinear effects enhancement^8–13^.

Typically, the proposed design methodologies can be categorized into two mechanisms, similar as those in the electromagnetics domain. In the realm of electromagnetics, there are two types of metamaterials: local resonances based metamaterials, such as the split-ring resonator and wire medium, and transmission line (TL) based metamaterials^14,15^. Materials of the first kind are inherently lossy and narrowband due to the nature of local resonances. The latter can achieve much larger bandwidth since they do not explicitly rely on resonance phenomena^16^. The investigation of acoustic metamaterials has followed the same path due to the similarity. In 2000, a locally resonant material was proposed that exhibited band gaps with the lattice constant in the deep-subwavelength scale^5^. The band gaps of the phononic crystals are induced by the local resonances of the structured unit cells. The unit cell is built with a solid sphere coated with soft silicon rubber and embedded into a hard matrix material and thus can be described by a spring mass model^17^. Being the mass, the solid sphere connected to the rigid matrix by the soft silicone rubber, which acts like a spring. Utilizing this lumped element method, resonance-induced negative effective bulk modulus and double-negative acoustic metamaterials were demonstrated theoretically and experimentally^18–21^. In contrast to the locally resonance based acoustic metamaterials, TL-based labyrinthine or path coiling acoustic metamaterials can provide broadband sound reduction and exhibit extremely high effective refractive index values due to their characteristic topological architecture. The first zigzag type labyrinthine metamaterial was proposed by Liang and Li^20^. The extreme effective properties of zig–zag-type labyrinthine metastructures result in a high impedance mismatch due to the geometry differences between channels. To address this issue, several different geometries such as helical, horn-like and spiral-like shapes^21–25^ have been proposed and all based on the impedance matching theory, which is very popular in the transmission line analysis. The ability of labyrinthine structures to reduce the amplitude of propagating waves due to multiple wave reflections within labyrinthine channels allowing almost perfect reflection of low-frequency airborne sound waves. By arbitrarily delaying the propagating phase along the curled channels, the phononic crystal can be constructed with bandgap structure at very low frequencies so that it can actually have a valid effective medium description with extreme constitutive parameters. This phase engineering approach can manipulate the refraction and reflection by generating the phase shift with coiled structure. Acoustic metasurfaces are constructed based on these path coiled unit cells. With these metasurfaces, acoustic mirrors, reflectors and lens^26,27^ were developed for sound reflection and amplification, just like their optical counterparts. Besides the advantage of broadband frequency range, labyrinthine path coiled structure is easy to fabricate when compared with local resonance based design, which involves complicated manufacturing and assembly process bringing by the different material inclusion. In addition, path coiled channels are naturally aligned with the thermal cooling design, which frequently utilizes the coiled shape fluid channels to increase the cooling surface.

This paper proposes an acoustic metasurface with path coiled structure to selectively manipulate the acoustic transmission on given surface. A design approach for the labyrinthine unit cells of acoustic metasurface is established to systematically achieve the controllable working frequency with required bandwidth. An impedance based analytical model is developed to theoretically calculate the transmission amplitude of unit cells with different geometries. Based on the theory, high pass and low pass unit cells are designed to verify the validity of the proposed theory.

## Results

### Design approach

The channels in the metamaterial unit cells can be designed with zigzag path to slow the acoustic wave propagating from one end to the other. This idea of coiling up space can be easily applied to three dimensions. The path-coiled channel can also be equivalent to an effective homogenous medium with extreme constitutive parameters as long as the dimension of the unit cells is much smaller than the wavelength of the background fluid passing through the channels. With this subwavelength channel widths assumption, only plane waves exist inside the channels. For transmission analysis, any geometrical shape of the zigzag coil path can be unfolded into straight channel with effective length *L*_*eff*_. Then the effective length is used to calculate the equivalent impedance and by solving the impedance network, we can derive the transmission amplitude. To analytically derive this acoustic transmission line, an equivalent pipe circuit impedance model can be used. Similar derivation can be found in Refs.^28–31^.

**Figure 1:**
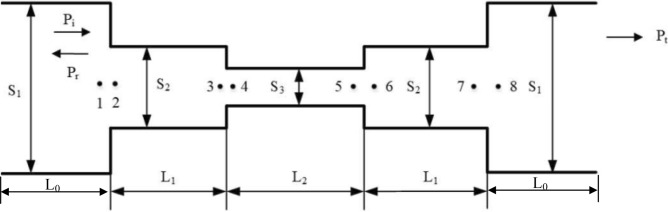
A labyrinthine metamaterial unit cell with unpacked equivalent straight channels.

A labyrinthine metamaterial unit cell with unpacked equivalent straight channels is shown in Fig. 1.

As mentioned above, the width of structure is smaller than half of acoustic wavelength, so only plane waves are allowed. For plane wave propagating in the above equivalent pipe channels, the acoustic pressure within the pipe is defined as

1 $$ p(x,t) = P_{ + } e^{j(\omega t - kx)} + P_{ - } e^{j(\omega t + kx)} $$

where *p* is the complex pressure (contains magnitude and phase), *x* is the location along the pipe starting at zero at the boundary between channels with different cross section width, *t* is time, *j* is *sqrt*(− 1), *ω* is the wave frequency, *k* is the wavenumber, and *P*_+_ and *P*_*−*_ are coefficients determined by the boundary conditions. The analytical solution of the transmission amplitude requires the impedance and pressure at each of the boundaries between two channels with different cross sectional width. Impedance calculations proceed from the outlet (point 8) to inlet (point 1) while pressure calculations proceed from inlet (point 1) to outlet (point 8). The impedance and pressure calculations start with the boundary conditions shown below

2 $$ \begin{aligned} & p_{n} = p_{n + 1} \quad n = 1,3,5,7 \\ & U_{n} = U_{n + 1} ,\quad Z_{n} = Z_{n + 1} \\ \end{aligned} $$

where *U* is the volume velocity and *Z* is the acoustic impedance.

The acoustic pressure is defined above in Eq. (1) and particle velocity is calculated as

3 $$ u(x,t) = \frac{{P_{ + } }}{\rho c}e^{j(\omega t - kx)} + \frac{{P_{ - } }}{\rho c}e^{j(\omega t + kx)} $$

The acoustic impedance down the path can be obtained by following the same methodology used above in a more generic form as

4 $$ z_{n} = \rho c\frac{{z_{n + 1} \cos (kL_{1} ) + j\rho c\sin (kL_{1} )}}{{\rho c\cos (kL_{1} ) - jz_{n + 1} \sin (kL_{1} )}}\quad n = 2,4,6 $$

In contrast to impedance calculations, pressure calculations proceed from inlet (point 1) to outlet (point 8). The acoustic pressure is represented in a more generic form as

5 $$ \, p_{n + 1} = \frac{{p_{n} }}{2}\left[ {\left( {1 + \frac{\rho c}{{z_{n} }}} \right)e^{{ - jkL_{n} }} + \left( {1 - \frac{\rho c}{{z_{n} }}} \right)e^{{jkL_{n} }} } \right] $$

As the outlet of the unit cell is anechoically terminated, the pressure at point 8 is simply the transmitted pressure *p*_*t*_. To meet the impedance matching condition, cross sectional width of the channels need to satisfy the following relation

6 $$ \, z_{2} = \sqrt {z{}_{1}z_{3} } \quad S_{2} = \sqrt {S{}_{1}S_{3} } $$

The final transmission amplitude can be obtained as

7 $$ \begin{aligned} T & = \left| {\frac{{p_{t} }}{{p_{i} }}} \right| = \frac{{2S_{1} S_{3} }}{{\sqrt {A^{2} + B^{2} } }} \\ A & = 2S_{1} S_{3} \cos^{2} (kL_{1} )\cos (kL_{2} ) - 2S_{1} S_{3} \sin^{2} (kL_{1} )\cos (kL_{2} ) \\ & \quad - 2\sqrt {S_{1} S_{3} } (S_{1} + S_{3} )\sin (kL_{1} )\cos (kL_{1} )\sin (kL_{2} ) \\ B & = - 2S_{1} S_{3} \sin^{2} (kL_{1} )\sin (kL_{2} ) + 2\sqrt {S_{1} S_{3} } (S_{1} + S_{3} )\sin (kL_{1} )\cos (kL_{1} )\cos (kL_{2} ) \\ & \quad + (S_{1}^{2} + S_{3}^{2} )\cos^{2} (kL_{1} )\sin (kL_{2} ) \\ \end{aligned} $$

**Figure 2:**
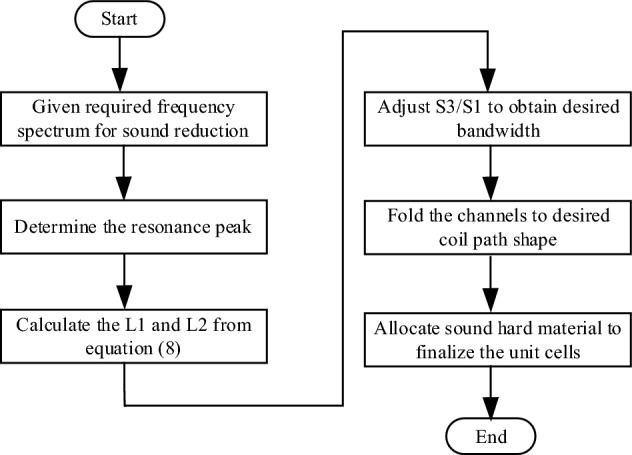
Design approach of labyrinth-based metasurface for noise reduction.

Considering the configuration of straight channels in Fig. 1, there are two different types of resonances having frequencies as

8 $$ \, f^{1} = \frac{(2n + 1)c}{{4L_{1} }}\quad n = 0,1,2 \ldots \quad f^{2} = 2f^{1} \quad f^{2} = \frac{nc}{{4L_{2} }}\quad n = 1,2,3 \ldots $$

with the first two resonance peak at $f_{0}^{1} { = }\frac{c}{{4L_{1} }}\quad f_{0}^{2} = \frac{c}{{4L_{2} }}$. The first type of resonance comes from the straight channel with effective length of *L*_*1*_, which is represented as pipe having one open-end and one close-end and thus has the resonance frequencies *f*^*1*^. The channel with effective length *L*_*2*_ can be modeled as pipe with two close-end, which has the resonance frequencies *f*^2^. The transmission amplitude in Eq. (7) depicts the sound pressure envelop with resonance peaks at frequencies shown in Eq. (8). The center frequency and bandwidth can be controlled by arranging the two resonance peak in the frequency domain and varying the cross sectional width ratio *S*_*3*_*/S*_*2*_. Once the effective length and cross sectional width of different channels are determined by the required center frequency and bandwidth, straight channels can then be folded into any shapes to form the unit cell of the acoustic metasurface materials. The abovementioned design approach is depicted in Fig. 2. In next section, a design example will be illustrated to show the practical design using the design approach depicted above.

### Design example

**Figure 3:**
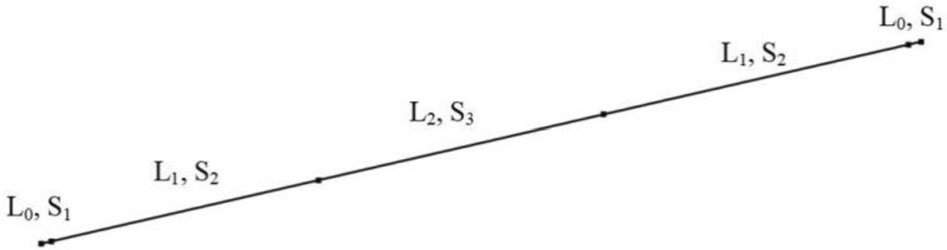
Equivalent pipe circuit with different segment length and cross-sectional areas.

In COMSOL, we use the pipe acoustic module to simulate the pipe circuit in 1D, as shown in Fig. 3, with different segment length and cross-sectional areas. In the design example illustrated in this section, the desired frequency gap is centered around 17,800 Hz with bandwidth of ± 200 Hz. The segment cross-sectional area ratio S_3_/S_1_ is initially determined and later optimized through the sensitivity analysis of its effect on bandgap width and shape.

**Figure 4:**
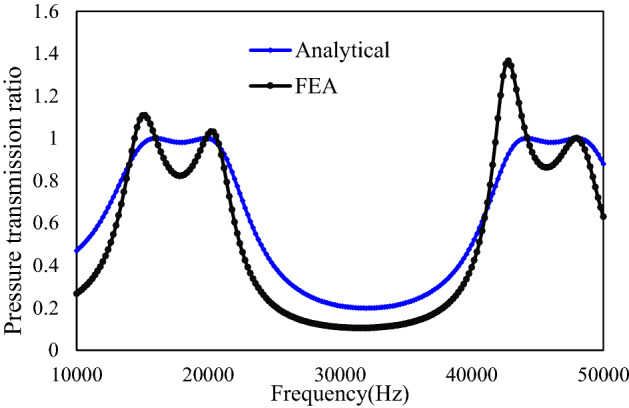
Comparison between equivalent pipe circuit analytical model and COMSOL simulation for straight pipe circuit.

The simulation is performed in COMSOL with the help of its acoustic pipe module. In simulation, anechoic termination is assumed to determine the end impedance of the pipe circuit. Under this assumption, the acoustic sound wave exits without any reflection. The simulation results are plotted in Fig. 1 overlay with the results from analytical calculation. In Fig. 4, the pressure transmission ratio is plotted versus the frequency. As can be seen from Fig. 4, the first and second resonance peaks center at round 17,800 Hz and 45600 Hz, which agrees with the desired design calculated from (8). Although there is some difference between the analytical calculation and simulation results, the bandgap shape accuracy is preserved. Thus, the equivalent pipe impedance based analytical model is valid for the bandgap design and optimization.

**Figure 5:**
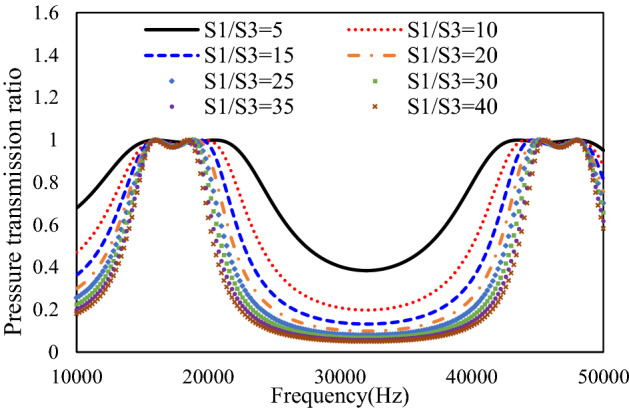
Sensitivity analysis of bandgap width with various cross-sectional area.

As can be seen from Fig. 4, the bandgap frequency characteristics from simulation and analytical model agree with each other although some inaccuracy exists. Thus, the proposed equivalent pipe circuit based analytical model is eligible for the initial design and optimization of the acoustic metamaterial unit cells. The length of the segment in unit cells determines the frequency resonance peaks while the cross-sectional ratio shapes the bandgap in between the resonance peaks. The length effect on resonance peak frequency is theoretically derived in (8) and validated by simulations. To evaluate the effect of cross-sectional areas on bandgap width, the bandgap characteristics are calculated from the proposed analytical model with various cross-sectional area S_3_ and fixed area S_1_. The cross-sectional area S_2_ is determined by the geometrical mean of S_1_ and S_3_ (S_2_ = sqrt(S_1_S_3_)) to serve the impedance matching purpose. The calculated results are plotted in Fig. 5.

It is found in Fig. 5 that higher cross-sectional area ratio widens the bandgap width. However, with the fixed cross-sectional area S_1_ at the entry of the unit metamaterial cell, the size of the narrowest cross section is constrained by the manufacturing tolerance. The entry area of the unit cells can be increased to enlarge the cross-sectional area ratio while still keeping the narrowest part within the manufacturing tolerance. The drawback is the increase of the unit cell size. Thus, a balance design among bandgap characteristics for noise reduction and manufacturing tolerance needs to be achieved.

**Figure 6:**
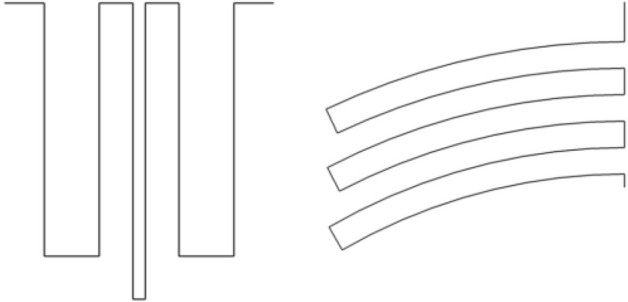
Two path coils with different shape (**a**) square shape path coils (**b**) radial circular shape path coils.

After dimension design of the straight pipe circuit, it is folded to desired path coil shape to form the unit cell of the metamaterials. It is assumed that the bandgap characteristics are preserved in this folding process as long as the length and cross-sectional areas of each segment are kept the same. To evaluate the validity of this assumption, two path coils with different shape having the same segment length and cross-sectional areas as the straight pipe circuit are modeled in COMSOL, as illustrated in Fig. 6.

**Figure 7:**
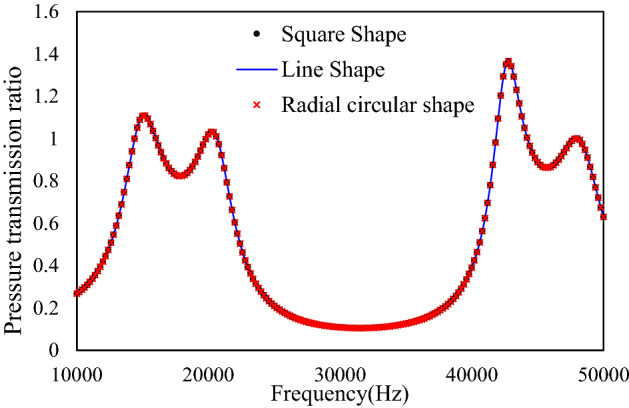
Simulation comparison for straight pipe circuit, square shape folded path coils and radial circular shape folded path coils.

Although the two path coils (a) and (b) have very different shape, the length and cross-sectional areas of the pipe are preserved. The simulations for both shapes have been performed in COMSOL and the results are plotted in Fig. 7.

The previous simulation result for the straight pipe circuit is plotted in the same figure for comparison. It is found that the bandgap characteristics are preserved for any shape of the path coils without considering the pipe losses due to the geometrical singularity. The critical design factors affecting the bandgap characteristics are the length and cross-sectional areas of each pipe segment. As long as they are fixed, we can design the labyrinth in any free form without deteriorating the noise reduction performance. Thus, the proposed metasurface is a generic design fulfilling wide range of dimensions.

**Figure 8:**
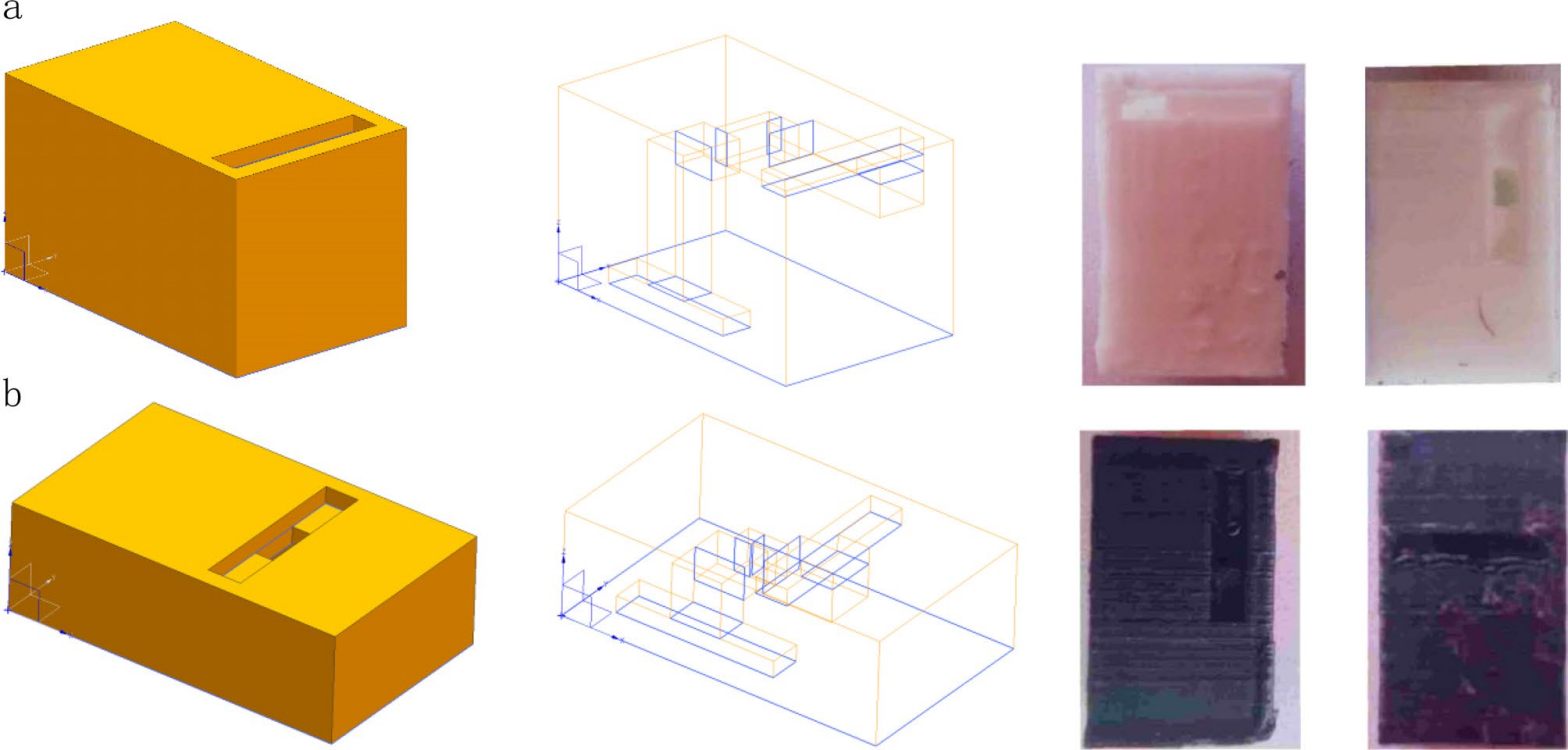
Unit cell of 3D modeling diagram and 3D print acoustic metamaterial. (**a**) High pass 3D modelling diagram and 3D printed unit cell. (**b**) Low pass 3D modelling diagram and 3D printed unit cell.

To demonstrate the design approach, an equivalent pipe circuit is designed with parameters shown in Table 1. The two different unit cells are designed in 3D domain shown in Fig. 8. In the design example illustrated in this section, the frequency of the acoustic transducer used is centered around 17,800 Hz. To meet the design requirement of high pass and low pass unit cells, the designed resonant frequency (*f*^1^ = 16,000 Hz forhigh pass unit cell and *f*^1′^ = 32,000 Hz for low pass unit cell) and the corresponding pipe length can be calculated based on Eq. (8).

**Table 1:** Geometrical and material parameters.

Dimensions	Value	Parameters	Value
*L*_0_	0.5 mm	Medium	Air
*L*_1_ (high pass)	5.36 mm	*c*	343 m/s
*L*_2_ (high pass)	2.68 mm	*L*_1_ (Low Pass)	2.68 mm
*L*_2_ (low pass)	1.34 mm	*S*_2_	1.58 mm^2^
*S*_1_	5 mm^2^	*S*_3_	0.5 mm^2^

**Figure 9:**
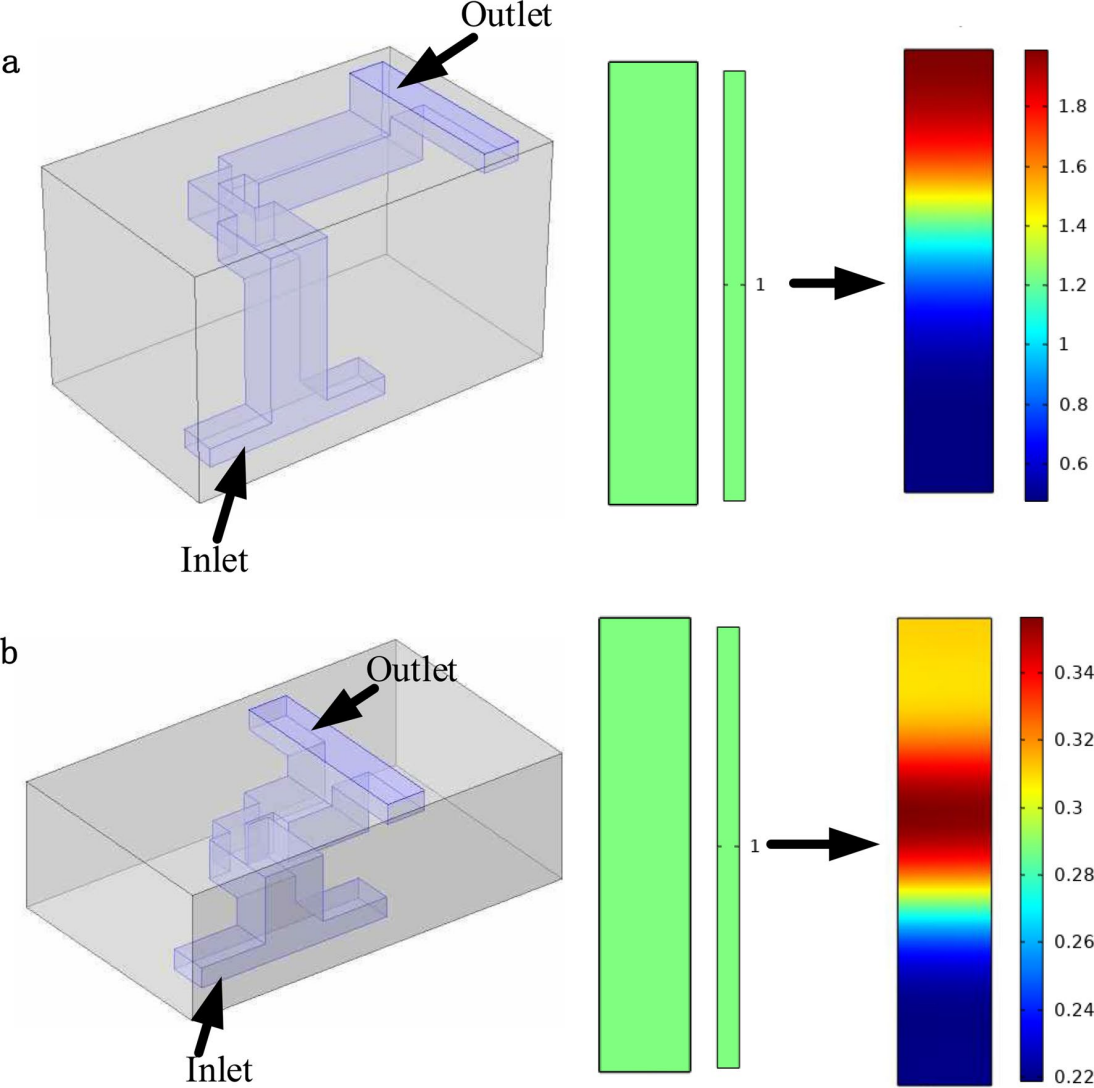
Acoustic pressure distribution at inlet and outlet of two designed unit cells. (**a**) Acoustic pressure distribution at outlet of high pass unit cell. (**b**) Acoustic pressure distribution at outlet of low pass unit cell.

The simulation is performed in COMSOL using its pressure acoustic module. The pipe wall is formed by material with moderate elastic modulus, while air is the working fluid flowing inside the pipe. The simulated acoustic pressure distribution at inlet and outlet of both unit cells is plotted at 17,800 Hz in Fig. 9. As shown in Fig. 9, the acoustic pressure at outlet has been noticebly enhanced when compared to pressure at inlet for high pass unit cell, which demonstrates that the transmission of sound wave energy is unencumbered. In contrast, the acoustic pressure at outlet of the low-pass unit cell decreased significantly, indicating that the metamaterial hindered the transmission of sound wave energy. The acoustic pressure ratio distribution between high-pass and low-pass unit cells at their outlets is about 1.3–5.

**Figure 10:**
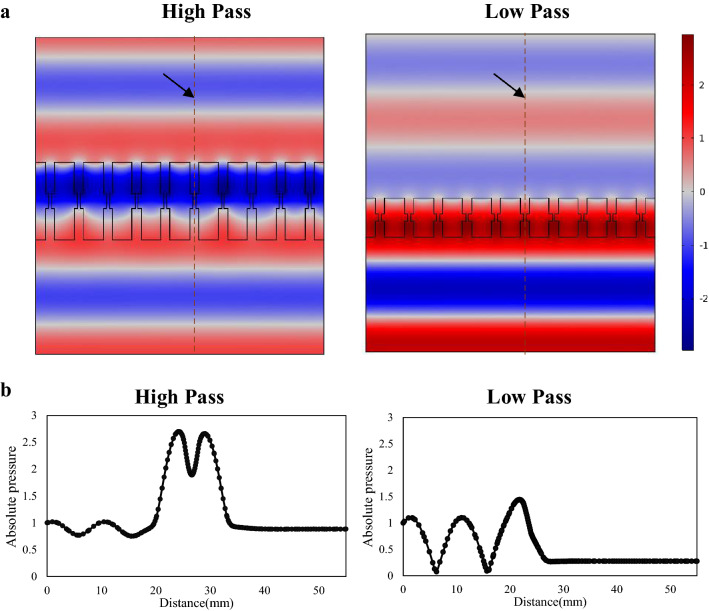
The simulation of the acoustic field. (**a**) The acoustic field distribution of high pass and low pass. (**b**) The acoustic absolute pressure distribution of high pass and low pass in the dash line of (**a**).

**Figure 11:**
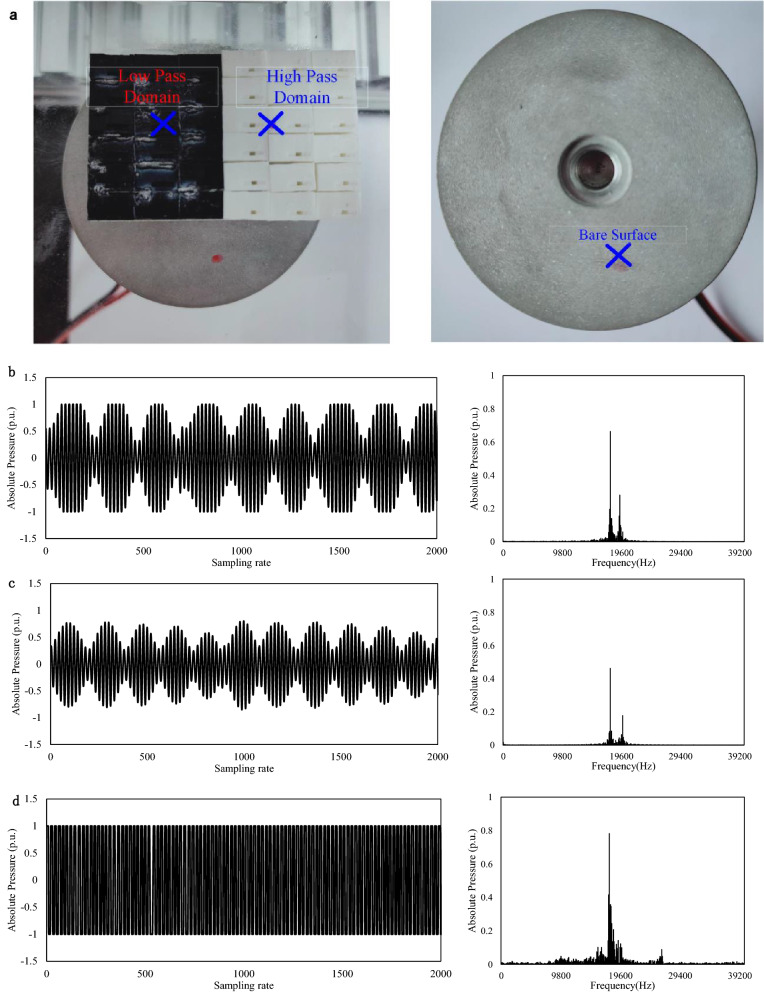
Experimental result. (**a**) Measurement location. (**b**) Experimental results and spatial harmonic distribution in high pass. (**c**) Experimental results and spatial harmonic distribution in low pass. (**d**) Experimental results and spatial harmonic distribution in high pass without barrier.

To further validate the simulation, the simulated 3D unit cells are 3D printed as acoustic LEGO blocks. The 3D printed high and low pass acoustic unit cells are then combined together to form the selective ultrasonic reflection metasurface. The configurable acoustic reflection metasurface is then used as ultrasonic acoustic reflectors for acoustic levitation demonstration. In order to clarify the acoustic field distribution under multiple high pass and low pass unit cells, we used COMSOL 2D to simulate the acoustic field distribution (see Fig. 10). The simulation adopts straight pipe based on the conclusion that the effect of pipe shape on sound field is almost negligible. The simulation results illustrate that the sound waves successfully pass through the high pass domain. Instead, sound waves are reflected by the low pass domain. Meanwhile, the acoustic pressure ratio distribution between high-pass and low-pass domain at their outlets is about 3.81 that is consistent with the conclusion in Fig. 9. Therefore, acoustic levitation can be realized in the low pass domain. The experimental results are shown in Fig. 11. Figure 11a shows the actual measured position by the microphone. In the figure, the black part is the low-pass domain, and the white part is the high-pass domain. To ensure the measurement accuracy, multiple cells of same type are combined together to form the separate low-pass and high-pass domain. In Fig. 5a, the surface in black is the low-pass domain while the surface in white is the high-pass domain. The position marked with ‘X’ in Fig. 11a is the microphone measurement location. Figure 11b–d show the normalized acoustic pressure waveforms with spatial harmonicsmeasured on high pass, low pass and bare surface above the ultrasonic transducer. As can be seen in Fig. 11, the acoustic pressure amplitude on high-pass domain (0.67) is higher than that on low-pass domain (0.44), which verifies the effect of acoustic metamaterials. The experimental ratio of high pass domain to low pass domain is about 1.5. It is consistent with the simulation results shown in Fig. 9.

**Figure 12:**
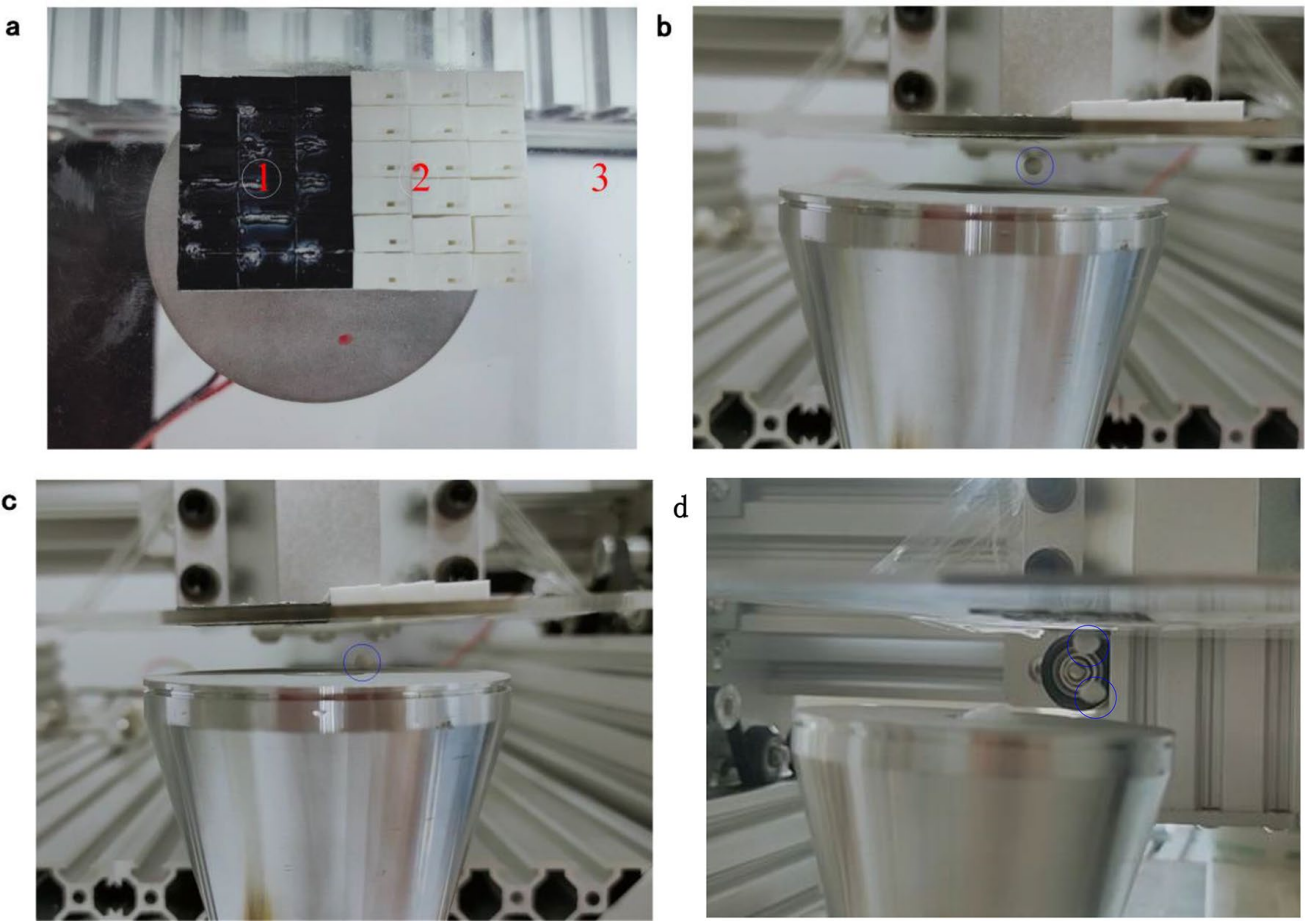
Acoustic levitation. (**a**) Acoustic levitation testing platform (**b**) Acoustic levitation in low pass domain (**c**) Acoustic levitation in high pass domain. (**d**) Acoustic levitation of two pellets in low pass domain.

The acoustic levitation experiment is designed to intuitively illustrate the properties of metamaterials. Figure 12a is the configuration of the testing platform. In the figure, ① represents low-pass domain. ② represents high-pass domain. ③ is PMMA frame mounting the low-pass and high-pass unit cells. Figure 12b is the experimental diagram of acoustic levitation in low pass domain. As shown in Fig. 12b, the foam plastic pellet is suspended in the air, indicating that the low-pass domain can reflect acoustic waves. As shown in Fig. 12c, the foam plastic pellet can not be suspended, which demonstrates that the acoustic waves can traverse high-pass domain and can not form standing waves that make the pellet levitate. Meanwhile, the distance between the upper and lower surfaces are expanded to achieve the levitation of two pellets (see Fig. 12d). The detailed levitation process can be found in the video in “Supplemental materials”.

**Figure 13:**
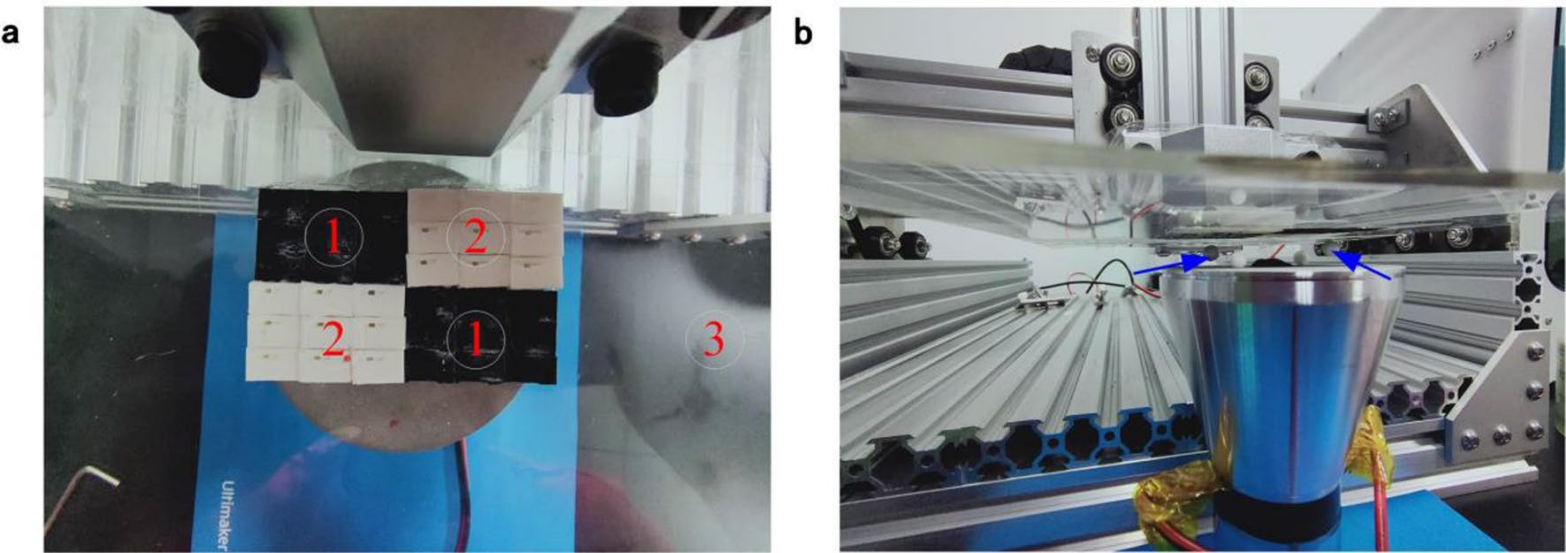
Acoustic resolution experiment. (**a**) Planform of acoustic resolution experiment platform. (**b**) Acoustic levitation.

In order to further demonstrate the properties of the acoustic metamaterials, acoustic metamaterial resolution experiment is designed. Figure 13a is the configuration of the acoustic metasurfacefor control resolution of acoustic levitation. As shown in Fig. 13b, the two small pellets under the low-pass region can be suspended, while the balls under the high-pass region cannot be suspended, illustrating the acoustic metamaterial resolution. Therefore, the technology can be used to arrange the suspended pellets into any shape.

## Discussion

This paper proposes a labyrinthine acoustic metasurface design approach with adjustable resonance peak frequencies and bandgap width in between. In this design approach, an equivalent pipe circuit based analytical model is used to predict the lossless transmission ratio for the straight pipe circuit with different segment length and cross-sectional area. From the analytical model, the resonance peak frequencies are determined by the length of channel segments and bandgap width in between can be adjusted by the cross-sectional area ratio. Based on the proposed design approach, the high pass and low pass unit cells are designed. The validity of the theoiry has been proved by simulations in COMSOL and experimental results. Furthermore, the experiments of acoustic levitation and its control resolution are designed to further validate the efficacy of the theory. The theory presented in this paper can serve as an efficient tool for metamaterial unit cells design and optimization for acoustic wave manipulation.

## Methods

### Acoustic experiment equipment

NX12 is used to desgin the 3D models of high-pass and low-pass unit cells. The corresponding high-pass and low-pass unit cell are 3D printed through the Ultimaker S3 3D printer. The materials used in the low pass unit cell is the Ultimaker black tough PLA. The materials used in the high pass unit cell is the Ultimake Clariant white PLA. The microphone is VT RTA-168A. The acoustic transducer is producted by KMDcsb in Shenzhen.

### Acoustic simulations

To simulate the transmission of the acoustic wave through the metamaterial unit cell layer, a finite element method (FEM)-based numerical simulation was conducted using COMSOL Multiphysics 5.5 with pressure acoustic frequency domain module.

## Supplementary Information

Supplementary Information 1.

Supplementary Video 1.

Supplementary Video 2.

Supplementary Video 3.

Supplementary Video 4.

Supplementary Video 5.
