# Supplementary material for: Design approach of perforated labyrinth-based acoustic metasurface for selective acoustic levitation manipulation
Source: Sci Rep. 2021 Apr 7;11:7619. doi: 10.1038/s41598-021-87179-x (PMC8027179; doi:10.1038/s41598-021-87179-x)
Supplement: Supplementary file 1 — Supplementary Information 1. [file 41598_2021_87179_MOESM1_ESM.pdf]

Design Approach of Perforated Labyrinth-based Acoustic Metasurface for Selective Acoustic Levitation Manipulation

Affiliations

1. **School of Electrical Engineering, Southeast University, Nanjing, Jiangsu, China**

   Zhike Xu, Ling Qin, Wei Xu*, Shuhua Fang

2. Ford motor company, Dearborn, MI, USA

   Jiyao Wang

Contributions

W. X. initiated the project. Z. X. and L. Q. performed the experimental demonstrations with assistance from W.X. and S. F.. J. W. performed the data analysis and simulation. All authors discussed the results and implications and commented on the paper at all stages. All authors wrote the paper and the Supplementary Information.

Corresponding author

*Correspondence to: Wei Xu [weixu@seu.edu.cn]

ORCID ID: https://orcid.org/0000-0002-7008-4364

Supplementary Information

File Name: Supplementary Movie 1

Description: Movie shows the process of foam plastic pellet acoustic levitation in low pass domain.

File Name: Supplementary Movie 2

Description: Movie shows that the foam plastic pellet can not levitate in high pass domain.

File Name: Supplementary Movie 3

Description: Movie shows the dynamic process of the floating state of the foam plastic pellet from the low pass domain to the high pass domain.

File Name: Supplementary Movie 4

Description: Movie shows the dynamic process of acoustic resolution experiment.

File Name: Supplementary Movie 5

Description: Movie shows the acoustic levitation of two pellets.

File Name: Supplementary Document

Description: The detailed deduction of the design approach is presented in supplementary document.

As mentioned above, the width of structure is smaller than half of acoustic wavelength, so only plane waves are allowed. For plane wave propagating in the above equivalent pipe channels, the acoustic pressure within the pipe is defined as

$$p(x,t) = P_{+}e^{j(\omega t - kx)} + P_{-}e^{j(\omega t + kx)} \tag{1}$$

where p is the complex pressure (contains magnitude and phase), x is the location along the pipe starting at zero at the boundary between channels with different cross section width, t is time, j is $sqrt(-1)$, ω is the wave frequency, k is the wavenumber, and P_+ and P_- are coefficients determined by the boundary conditions. The analytical solution of the transmission amplitude requires the impedance and pressure at each of the boundaries between two channels with different cross sectional width. Impedance calculations proceed from the outlet (point 8) to inlet (point 1) while pressure calculations proceed from inlet (point 1) to outlet (point 8). The impedance and pressure calculations start with the boundary conditions shown below

$$\begin{aligned} &p_n = p_{n+1} \quad n=1,3,5,7 \\ &U_n = U_{n+1}, \ Z_n = Z_{n+1} \end{aligned} \tag{2}$$

where U is the volume velocity and Z is the acoustic impedance.

The specific acoustic impedance at outlet (point 8) is equal to the characteristic impedance with anechoic termination assumption, where ρ is density of the background fluid, c is speed of sound in the background fluid. Thus the acoustic impedance at outlet (point 8) is given by

$$Z_8 = \frac{z_8}{S_1} = \frac{\rho c}{S_1} \tag{3}$$

where S_1 is the cross sectional width at outlet (point 8). From boundary conditions in equation (2), the acoustic pressures and volume velocities at point 7 and 8 are equal. Hence, the acoustic impedance at these two points are equal. Thus, the specific acoustic impedance at point 7 can be derived as

$$z_7 = Z_7 \cdot S_2 = Z_8 \cdot S_2 = z_8 \cdot \frac{S_2}{S_1} \tag{4}$$

The specific impedance at point 6 can be further found with the plane wave propagating in the channel between point 6 and 7 using equivalent pipe model. The acoustic pressure is defined above in equation (1) and particle velocity is calculated as

$$u(x,t)=\frac{P_+}{\rho c}e^{j(\omega t-kx)}+\frac{P_-}{\rho c}e^{j(\omega t+kx)} \tag{5}$$

The specific acoustic impedance can be further derived using the pressure and particle velocity as

$$z_7=\frac{p_7(x=L_1)}{u_7(x=L_1)}=\rho c(\frac{P_+e^{j(\omega t-kL_1)}+P_-e^{j(\omega t+kL_1)}}{P_+e^{j(\omega t-kL_1)}-P_-e^{j(\omega t-kL_1)}})=\rho c[\frac{P_+e^{-jkL_1}+P_-e^{jkL_1}}{P_+e^{-jkL_1}-P_-e^{-jkL_1}}] \tag{6}$$

Equation (6) can be rearranged to give the plane wave coefficients

$$P_+=\frac{z_7+\rho c}{z_7-\rho c}\cdot P_-e^{2jkL_1} \tag{7}$$

Similarly, at $x = 0$ (corresponding to point 6 in Figure 1), the specific acoustic impedance can be found as function of specific acoustic impedance at point 7

$$z_6=\frac{p_6(x=0)}{u_6(x=0)}=\rho c(\frac{P_++P_-}{P_+-P_-})=\rho c[\frac{(z_7+\rho c)e^{jkL_1}+(z_7-\rho c)P_-e^{-jkL_1}}{(z_7+\rho c)e^{jkL_1}-(z_7-\rho c)P_-e^{-jkL_1}}] \tag{8}$$

which may be further simplified in non-complex form as

$$z_6=\rho c\frac{z_7\cos(kL_1)+j\rho c\sin(kL_1)}{\rho c\cos(kL_1)-jz_7\sin(kL_1)} \tag{9}$$

The acoustic impedance down the path can be obtained by following the same methodology used above in a more generic form as

$$z_n=\rho c\frac{z_{n+1}\cos(kL_1)+j\rho c\sin(kL_1)}{\rho c\cos(kL1)-jz_{n+1}\sin(kL_1)} \quad n=2,4,6 \tag{10}$$

In contrast to impedance calculations, pressure calculations proceed from inlet (point 1) to outlet (point 8). At point 1 in Figure 1, the pressure and specific acoustic impedance are given by

$$p_1=p_i+p_r,\quad z_1=\rho c(\frac{p_i+p_r}{p_i-p_r}) \tag{11}$$

where p_i is the pressure of incident wave and p_r is pressure of reflected wave. Equation (11) can be rearranged to derive the acoustic pressure at point 1

$$p_1 = \frac{2p_i}{(1+\rho c / z_1)} \tag{12}$$

From boundary conditions in Equation (2), equality of the acoustic pressure at point 1 and 2 leads to $p_2 = p_1$, x=0. Similar as impedance at point 6 in Equation (9), the specific acoustic impedance at point 2 can be calculated as

$$z_2 = \frac{p_2(x=0)}{u_2(x=0)} = \rho c\left(\frac{P_+ + P_-}{P_+ - P_-}\right) \tag{13}$$

and plane wave pressure at point 2 is given as

$$p_2 = p(x=0,t) = P_+ e^{j\omega t} + P_- e^{-j\omega t} \tag{14}$$

Pressure wave coefficients in the channel between point 2 and 3 can be derived by rearranging the equation (13) and (14)

$$P_+ = \frac{p_2(z_2+\rho c)}{2z_2} e^{-j\omega t}, P_- = \frac{p_2(z_2+\rho c)}{2z_2} e^{-j\omega t} \tag{15}$$

Inserting (15) back to the plane wave equation, the pressure at point 3 is obtained as

$$p_3 = p(x=L_1,t) = P_+ e^{j\omega t\text{-}kL_1} + P_- e^{-j(\omega t+kL_1)} = \frac{p_2}{2}\left[(1+\frac{\rho c}{z_2})e^{-jkL_1} + (1\text{-}\frac{\rho c}{z_2})e^{jkL_1}\right] \tag{16}$$

The acoustic pressure at point 3 to 8 in response to incident wave of pressure p_i can be derived by following the same methodology in the development of equation (16), which is represented in a more generic form as

$$p_{n+1} = \frac{p_n}{2}\left[(1+\frac{\rho c}{z_n})e^{-jkL_n} + (1\text{-}\frac{\rho c}{z_n})e^{jkL_n}\right] \tag{17}$$

As the outlet of the unit cell is anechoically terminated, the pressure at point 8 is simply the transmitted pressure p_t. To meet the impedance matching condition, cross sectional width of the channels need to satisfy the following relation

$$z_2 = \sqrt{z_1 z_3} \quad S_2 = \sqrt{S_1 S_3} \tag{18}$$

Combining all the equations above, the final transmission amplitude can be obtained as

$$\begin{aligned} T &= \left|\frac{p_t}{p_i}\right| = \frac{2S_1S_3}{\sqrt{A^2+B^2}} \\ A &= 2S_1S_3\cos^2(kL_1)\cos(kL_2) - 2S_1S_3\sin^2(kL_1)\cos(kL_2) \\ &\quad -2\sqrt{S_1S_3}(S_1+S_3)\sin(kL_1)\cos(kL_1)\sin(kL_2) \\ B &= -2S_1S_3\sin^2(kL_1)\sin(kL_2) + 2\sqrt{S_1S_3}(S_1+S_3)\sin(kL_1)\cos(kL_1)\cos(kL_2) \\ &\quad +(S_1^2+S_3^2)\cos^2(kL_1)\sin(kL_2) \end{aligned} \tag{19}$$

Considering the configuration of straight channels in Figure 1, there are two different types of resonances having frequencies as

$$f^1 = \frac{(2n+1)c}{4L_1} \quad n=0,1,2... \quad f^2 = 2f^1 \quad f^2 = \frac{nc}{4L_2} \quad n=1,2,3... \tag{20}$$

with the first two resonance peak at $f_0^1 = \frac{c}{4L_1} \quad f_0^2 = \frac{c}{4L_2}$. The first type of resonance comes from the straight channel with effective length of L_1, which is represented as pipe having one open-end and one close-end and thus has the resonance frequencies f^1. The channel with effective length L_2 can be modeled as pipe with two close-end, which has the resonance frequencies f^2. The transmission amplitude in equation (19) depicts the sound pressure envelop with resonance peaks at frequencies shown in equation (20). The center frequency and bandwidth can be controlled by arranging the two resonance peak in the frequency domain and varying the cross sectional width ratio S_3/S_2. Once the effective length and cross sectional width of different channels are determined by the required center frequency and bandwidth, straight channels can then be folded into any shapes to form the unit cell of the acoustic metasurface materials.
